# Peer-led healthy lifestyle program in supportive housing: study protocol for a randomized controlled trial

**DOI:** 10.1186/s13063-015-0902-z

**Published:** 2015-09-02

**Authors:** Leopoldo J. Cabassa, Ana Stefancic, Kathleen O’Hara, Nabila El-Bassel, Roberto Lewis-Fernández, José A. Luchsinger, Lauren Gates, Richard Younge, Melanie Wall, Lara Weinstein, Lawrence A. Palinkas

**Affiliations:** Columbia University School of Social Work, 1255 Amsterdam Avenue, New York, NY 10027 USA; New York State Psychiatric Institute, 1051 Riverside Drive, New York, NY 10032 USA; Columbia University Medical Center, Presbyterian Hospital, 622 West 168th St, New York, NY 10032 USA; New York Presbyterian, Family Medicine, 610 West 158th St, New York, NY 10032 USA; Jefferson University Hospital, 833 Chestnut Street, Philadelphia, PA 19107 USA; University of Southern California School of Social Work, University Park, Los Angeles, CA 90089 USA

**Keywords:** Hybrid design, Serious mental illness, Healthy lifestyle interventions, Health disparities, Implementation science, Effectiveness trial, Obesity

## Abstract

**Background:**

The risk for obesity is twice as high in people with serious mental illness (SMI) compared to the general population. Racial and ethnic minority status contribute additional health risks. The aim of this study is to describe the protocol of a Hybrid Trial Type 1 design that will test the effectiveness and examine the implementation of a peer-led healthy lifestyle intervention in supportive housing agencies serving diverse clients with serious mental illness who are overweight or obese.

**Methods:**

The Hybrid Trial Type 1 design will combine a randomized effectiveness trial with a mixed-methods implementation study. The effectiveness trial will test the health impacts of a peer-led healthy lifestyle intervention versus usual care in supportive housing agencies. The healthy lifestyle intervention is derived from the Group Lifestyle Balanced Program, lasts 12 months, and will be delivered by trained peer specialists. Repeated assessments will be conducted at baseline and at 6, 12, and 18 months post randomization. A mixed-methods (e.g., structured interviews, focus groups, surveys) implementation study will be conducted to examine multi-level implementation factors and processes that can inform the use of the healthy lifestyle intervention in routine practice, using data from agency directors, program managers, staff, and peer specialists before, during, and after the implementation of the effectiveness trial.

**Discussion:**

This paper describes the use of a hybrid research design that blends effectiveness trial methodologies and implementation science rarely used when studying the physical health of people with SMI and can serve as a model for integrating implementation science and health disparities research. Rigorously testing effectiveness and exploring the implementation process are both necessary steps to establish the evidence for large-scale delivery of peer-led healthy lifestyle intervention to improve the physical health of racial/ethnic minorities with SMI.

**Trial registration:**

www.clinicaltrials.gov; NCT02175641, registered 24 June 2014

## Background

Obesity and related conditions (e.g., type-2 diabetes (T2D)) disproportionately impact people with serious mental illness (SMI: e.g., schizophrenia) compared to the general population [1–4]. Racial/ethnic minority status confers additional risks. Compared to non-Latino whites with SMI, Latinos [4–9] and African Americans [10] with SMI are at higher risk of obesity, metabolic syndrome, and T2D. Modifiable risk factors for obesity (e.g., physical inactivity) are more prevalent among low-income African Americans and Latinos than whites [11] and are key contributors of excess mortality among people with SMI [12–14]. Regardless of race/ethnicity, addressing obesity and overweight in people with SMI is critical, as these health conditions adversely affect treatment adherence, relapse, quality of life, and are linked to premature mortality [2].

Healthy lifestyle interventions can combat overweight/obesity and lower the risk of T2D and cardiovascular disease (CVD). The Group Lifestyle Balance (GLB), a healthy lifestyle intervention derived from the Diabetes Prevention Program, has proven efficacious for preventing and delaying T2D and CVD in the general population through its impact on weight loss [15–20]. GLB is an evidence-based intervention delivered in groups that uses behavioral strategies (e.g., self-monitoring) to support healthy diet modifications and increase physical activity [21]. Among people with SMI, healthy lifestyle interventions can achieve improvements in body weight, body mass index (BMI), cardiometabolic indicators, and quality of life, particularly if they focus on multiple lifestyle factors and use behavioral strategies [22–25].

Recent studies show that when lifestyle interventions are accessible to people with SMI in clinical settings, they can result in clinically significant weight loss [26, 27]. For example, participants in the ACHIEVE trial who received an 18-month behavioral weight-loss intervention in outpatient psychiatric rehabilitation programs lost 7 lbs on average and 37.8 % lost ≥ 5 % of their initial weight, compared to 22.7 % of controls [27]. Despite these results, healthy lifestyle interventions are usually delivered in clinical settings by clinical staff and are rarely available in other community settings that serve people with SMI (e.g., housing agencies) due to logistical, access, and staffing barriers. The effectiveness of healthy lifestyle interventions among racial/ethnic minorities with SMI living in the community also remains unclear. In a systematic literature review of lifestyle interventions for people with SMI in the United States (US), minorities represented less than one fourth of study participants and only one study (*n* = 8) included Spanish-speaking Hispanics [22]. More research has been done recently, but only in clinical settings. A study tested the acceptability and feasibility of a weight loss program among 43 Latino patients with SMI in an outpatient clinic [28], and the ACHIEVE trial sample was 38 % black. More work is needed given the growth of the national racial/ethnic minority population and the persistent health disparities they face.

### Peer-led healthy lifestyle intervention for people with SMI

Healthy lifestyle interventions, like GLB, can produce health benefits for people with SMI as they target modifiable risk factors linked to T2D and CVD [29, 30]. A key focus of GLB is to achieve ≥ 5 % weight loss, which yields reductions in morbidity and mortality from T2D and CVD in people who are overweight or obese. This level of weight loss raises HDL (“good”) cholesterol and lowers total and LDL (“bad”) cholesterol, triglycerides, glucose, and blood pressure [31–33]. GLB also leads to increased physical activity to the recommended level of ≥ 150 minute/week of moderate activity (e.g., brisk walking) [34]. This threshold reduces cardiometabolic risk independent of weight loss [35] and is linked to a 14 % reduction in risk for all-cause mortality [36]. Given these health benefits, GLB is being translated to communities impacted by the obesity and T2D epidemics [37, 38]. A meta-analysis of 28 US-based effectiveness studies in the general population examining the impact of GLB programs found that the average weight loss at 12 months was 4–5 % and that this outcome was achieved regardless of whether GLB was delivered by trained professionals or community health workers in a group format [37]. These findings suggest that moving GLB to community settings serving people with SMI by using a group format and trained peer specialists is an important next step that can make GLB more accessible and economically feasible without diluting its effectiveness.

Peer specialists are individuals that have personal experiences with health/mental health issues who have progressed in their recovery and received training to support others. They are a growing segment of the mental health workforce [39] with more than 30 states in the US having some level of Medicaid reimbursement for peer specialists, and this number will grow with the implementation of the Affordable Care Act [40, 41]. Peer-led interventions have been successfully used in other health conditions [42–46]. Peer specialists bring credibility, trust, and hope to people with SMI [47]. Peer specialists can expose clients to positive and credible role models who can tap into their own experiences to provide instrumental, informational, and emotional support; help translate the health intervention into clients’ daily activities on ecological and cultural terms; and become credible “coaches” [48, 49].

Peer-led programs for people with SMI tend to produce as good or better results than non-peer-led programs, particularly when peer specialists deliver evidence-based interventions. Druss et al. [48] found that a peer-led self-management program derived from a chronic illness self-management intervention [42] was superior to usual care (UC) at 6 months in producing significant improvements in patients’ activation, primary care visits, and physical health-related quality of life. Although these results are promising, peer-led interventions have not targeted modifiable healthy lifestyle factors that can reduce obesity and prevent T2D and CVD. Establishing the effectiveness of Peer GLB in community settings, like supportive housing, is a logical next step that requires rigorous evaluation.

### Why deliver healthy lifestyle interventions in supportive housing?

Supportive housing refers to programs that provide access to affordable community-based housing and flexible services to address clients’ health and psychosocial needs [49]. Supportive housing units account for the largest share (39 %) of the housing inventory dedicated to housing the homeless in the US [50]. Approximately 80 % of supportive housing units in the US serve people with SMI, and this number is growing [51]. Supportive housing agencies are an ideal setting to deliver healthy lifestyle programs for several reasons. First, delivering Peer GLB at these agencies addresses access barriers as it brings a health intervention to people’s doorsteps [49]. Second, given the expansion of services supported by the Affordable Care Act, many supportive housing agencies are moving towards integrating health interventions into their operations [52, 53]. Third, these agencies already deliver group-based services (e.g., vocational classes). Fourth, these agencies serve people with a range of psychiatric diagnoses and health conditions, thus interventions delivered in these settings will have a broad reach among the SMI population. Fifth, many supportive housing agencies employ peer specialists; therefore, a peer-led healthy lifestyle intervention provides an economically feasible approach that fits with their existing staff. Last, we have found that clients have strong preferences for bringing peer-led healthy lifestyle interventions into these agencies’ settings [54].

### Conceptual framework

Hybrid designs take on “a dual focus in assessing clinical effectiveness and implementation bridging the gap between these two areas of research” [55]. A Hybrid Trial Type 1 design, as proposed in this study, tests the effects of a clinical intervention on client-level outcomes, while exploring multi-level implementation factors that can inform the use of the intervention in real-world settings. Studying how community agencies implement an established intervention, even within an effectiveness study, can advance the translation of research into practice in several ways. It provides an opportunity to explore longitudinally how the introduction of a new program influences and is influenced by agencies’ context. It can help identify the structures, policies, organizational characteristics, and processes that facilitate or impede the use of peer-led health interventions in community settings. This identification is critical, as staff attitudes and organizational practices can undermine the integration of peer-led services within organizations [56–58]. Finally, a hybrid design can generate knowledge necessary to develop implementation strategies that can accompany these interventions to enhance their use in routine practice.

**Fig. 1:**
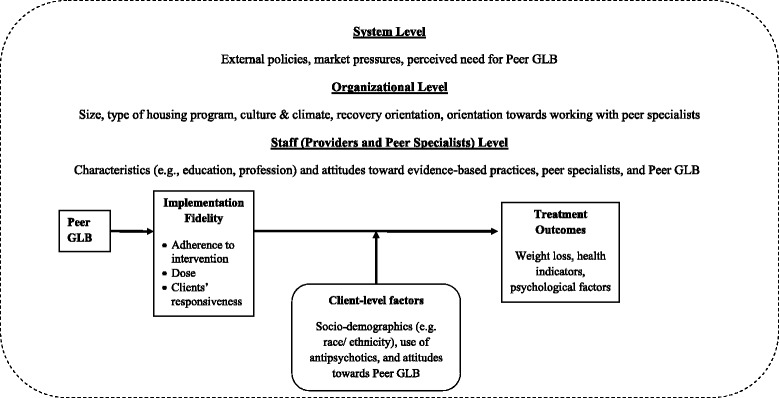
Conceptual framework for effectiveness and implementation research

Our study is guided by a multi-level framework (see Fig. 1) that draws from different implementation theories [59–61] and posits that the effectiveness of Peer GLB in supportive housing client-level outcomes will be mediated by the fidelity with which the intervention is delivered and moderated by several client-level factors. This entire process occurs within a context composed of system-level, organizational-level, and staff-level factors known to influence the implementation of evidence-based practices [61].

### Implementation fidelity

Fidelity is an implementation outcome that captures the degree to which an intervention is delivered as prescribed [59, 62]. In this study, we focus on three fidelity elements: 1) adherence: the degree to which components of Peer GLB are delivered; 2) dose: the number and type of Peer GLB sessions delivered; 3) participants’ responsiveness: the extent to which participants engages and are satisfied with Peer GLB. Attention to these fidelity elements has been recommended for implementation research [63] and will allow us to examine the extent to which peer specialists deliver Peer GLB as specified in our protocol.

### Client-level factors

Our model stipulates that client-level factors will moderate the effectiveness of Peer GLB. Data from the Diabetes Prevention Program indicate that black women in the lifestyle intervention group lost significantly less weight than other racial/ethnic and gender groups [64]. The use of antipsychotic medications that cause weight gain can also attenuate weight loss [2]. In contrast, clients’ positive attitudes toward Peer GLB (e.g., satisfaction) can result in better treatment engagement and outcomes.

### System-level factors

The delivery of Peer GLB is embedded within a multi-level context [61]. At the system level, we include factors beyond supportive housing that affect Peer GLB implementation, such as reimbursement policies and regulations to employ peer specialists, market pressures due to the Affordable Care Act, reimbursement policies and incentives for wellness programing, and agencies’ leaders’ perceived need for Peer GLB. We explore these system-level factors in our implementation study as they can help identify key policies and resources to leverage the implementation of health interventions in community settings serving people with SMI.

### Organizational-level factors

At the organizational level, we capture the inner context of housing agencies, such as the organizations’ characteristics and social context that influence the implementation, quality, and outcomes of evidence-based practices [60, 61]. We follow Glisson et al. [60] in their conceptualization of organizational social context composed of three interrelated domains: culture, climate, and work attitudes. Organizational culture refers to the values and norms that govern how work is done in an organization and is characterized as rigid, proficient or resistant [65]. Organizational climate refers to employee perceptions of the psychological impact of the work environment on their well-being and functioning in the organization and is characterized as engaged, functional, or stressful [60]. Work attitudes include the employees’ affective attachments to their jobs and encompass job satisfaction and commitment to the organization [65]. We include agencies’ recovery orientation [66] as one of our organizational factors given the link between health and recovery [67]. We capture factors that influence the integration of peer-led services within organizations, such as staff attitudes toward peer specialists, role conflicts, and supervision [56–58]. Understanding how organizational-level factors shape Peer GLB’s implementation is critical as implementation occurs in organizations and their contexts shape how providers adopt new practices [68]. Many organizational factors are also malleable through strategic interventions.

### Staff-level factors

At the staff level, we focus on staff characteristics and attitudes towards evidence-based practices (EBPs) and Peer GLB, as they influence the implementation of practice innovations. Attitudes toward EBPs consist of four interrelated dimensions: intuitive appeal of EBPs, likelihood of adopting EBPs if required by the organization, general openness to learning new practices, and perceived divergence between research-based practices and clinical experiences [65, 69]. We draw on the work of Rogers [70] and Damschroder et al. [61] to examine staff attitudes toward Peer GLB by focusing on key intervention characteristics (e.g., relative advantage) that influence implementation decisions [70]. Examining how staff-level factors impact and are impacted by the implementation of Peer GLB is critical as implementation is driven by staff behaviors that are influenced by staff characteristics, attitudes, intentions, and motivations. Staff-level factors are also shaped by organizational factors (e.g., culture) and represent a set of proximal determinants of providers’ and peer specialists’ willingness to adopt new interventions [65, 68]. Taken together, our model will serve as a guiding framework integrated in all stages of the research process.

### Study aims

1. Compare the effectiveness of Peer GLB versus UC in achieving clinically significant weight loss (≥5 % weight loss) at 12 and 18 months among overweight/obese (BMI ≥ 25) clients in supportive housing agencies.
2. Compare reductions in total weight, waist circumference, blood pressure, and improvements in physical activity, self-efficacy, recovery, and quality of life at 6, 12, and 18 months post randomization between Peer GLB and UC.
3. Explore whether client-level characteristics (e.g., socio-demographics, antipsychotic use, attitudes toward Peer GLB) moderate Peer GLB’s effectiveness.
4. Identify system-level, organizational-level, and staff-level factors that facilitate or impede the implementation of Peer GLB in supportive housing agencies.

## Methods

### Overview of hybrid design

**Fig. 2:**
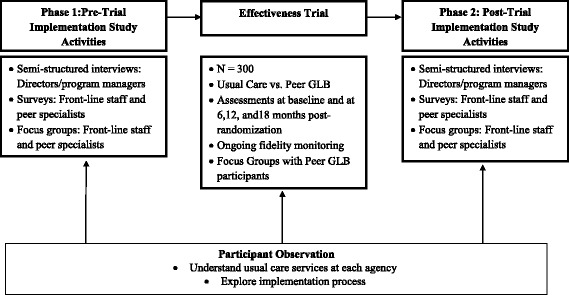
Hybrid Trial Type 1 design

We use a Hybrid Trial Type 1 design (see Fig. 2) that combines a randomized effectiveness trial with a mixed-methods implementation study. The study has been approved by the Institutional Review Boards of Columbia University and the Philadelphia Department of Health.

### Study sites

Three supportive housing agencies will serve as our community partners. The first agency uses a *Housing First* model, a SAMHSA-recognized EBP in which clients are offered an apartment throughout the community without requiring them to participate in formal treatment [71, 72]. By contrast, the other two agencies follow a treatment-first model in which clients are offered housing in either buildings that they manage or in apartments throughout the community, on the condition that they receive mental health services. The differences in these agencies enhances the wider reach and applicability of Peer GLB as it enables us to examine how to translate this intervention in two widely used supportive housing models.

### Randomized effectiveness trial (Aims 1–3)

#### Recruitment

Trained bilingual research assistants (RAs) will recruit participants through flyers, word-of-mouth, staff referrals, and presentations at groups and social events at each study site. For interested participants, RAs will conduct a face-to-face screening interview to determine study eligibility, and for those eligible, obtain informed consent. Eligibility criteria (Table 1) are similar to the ones used in previous weight loss trials [27, 28] and are broad in order to capture a sample that resembles racially/ethnically diverse people with SMI served by supportive housing agencies. RAs will then conduct baseline interviews face-to-face with eligible participants at their site within a week of recruitment.

**Table 1:** Eligibility criteria for effectiveness trial

Inclusion criteria	
Demographics	Male or female, 18 years of age or older, of any race/ethnicity, who are English and/or Spanish speakers and are receiving supportive housing services at the study sites
Mental health diagnosis	Chart diagnosis of a serious mental illness (e.g., schizophrenia, bipolar disorder, schizoaffective disorders, major depression)
Body mass index	BMI ≥ 25 (kg/m^2^)
Informed consent	Able and willing to give written informed consent to participate in study
Willingness to obtain medical clearance	Able and willing to obtain a medical clearance letter from a primary care physician with assistance from the study team if randomly assigned to the Peer GLB group
Exclusion criteria	
Substance abuse	Need for detoxification services at the time of recruitment
Suicidal/Homicidal ideation	Pose a danger to self or others at the time of recruitment
Capacity to consent	Failed capacity-to-consent questionnaire [88]
Cognitive impairment	For participants 65 years or older: screen positive for cognitive impairment based on Mini-cog clock test [89]
Medical contraindications to participate in weight loss program	Self-report of any of the following medical conditions that are contraindicated to participation in a weight loss program: cancer requiring active treatment, liver failure, history of anorexia nervosa, cardiovascular event (e.g., unstable angina, myocardial infarction) within the past 6 months, walking limitations preventing participation in exercise, history of weight loss surgery or planning weight loss surgery during study period, and (for female participants) pregnant or planning a pregnancy during study period.
	(Exclusion criteria for participants randomized to the intervention group: primary care physician confirms that the person has any of the following medical conditions: cancer requiring active treatment, liver failure, history of anorexia nervosa, cardiovascular event (e.g., unstable angina, myocardial infarction) within the past 6 months, walking limitations preventing participation in exercise, history of weight loss surgery or planning weight loss surgery during study period, and (for female participants) pregnant or planning a pregnancy during study period

*GLB* Group Lifestyle Balance

### Randomization

A total of 300 clients will be recruited and randomized to a single-blind randomized effectiveness trial. Within each of the 3 sites, participants will be randomized 1:1 to Peer GLB or UC, resulting in approximately 50 clients randomized to Peer GLB and 50 to UC within each site. Peer GLB groups at each site will begin once 6–8 new people have been randomized to Peer GLB.

### Intervention condition

GLB is a 12-month group-based healthy lifestyle intervention adapted from the original Diabetes Prevention Program lifestyle intervention [73] by the Diabetes Prevention Support Center at the University of Pittsburgh (see http://www.diabetesprevention.pitt.edu/ for more details about the GLB program). GLB is a goal-based behavioral intervention to achieve and maintain 7 % weight loss through modest dietary restrictions and moderate physical activity up to 150 minutes/week [20]. GLB consists of weekly core group sessions (3 months), bi-monthly transitional group sessions (3 months), and monthly maintenance sessions (6 months) delivered in community settings. Key features include integration of safe and appropriate nutrition education, physical activity recommendations, and behavioral change strategies including the use of self-monitoring tools (e.g., physical activity logs) with regular feedback, and the incorporation of problem-solving techniques to reduce barriers to healthy lifestyles. GLB includes a group facilitator manual and participant handouts for each session available in English and Spanish.

Peer specialists will be hired at each study site to deliver the Peer GLB intervention. Peer specialists must have the following qualifications: GED/high school diploma, lived experience managing an SMI, and completion of a peer specialist training program (e.g., Howie the HARP Program in New York City). Peer specialist training will include: 1) a 2-day GLB workshop for peer specialists and their supervisors to learn how to deliver the intervention; 2) twice a week post-workshop training sessions for up to 3 months for peer specialists to experience an abridged version of GLB; 3) a workforce development workshop to address issues of integrating peer specialists into agency staff; and 4) mock practice groups facilitated by peer specialists. During these mock sessions, the study team will provide peer specialist feedback on their group facilitation skills, knowledge of the intervention techniques, and competence completing intervention tasks. We will also conduct professional development workshops 2–3 times a year during the trial to address any emerging issues related to the integration of peer specialists into the workforce at each study site.

### Usual care condition

Study sites offer services to help clients with their physical health through health promotion groups, linkages to medical care, and community resources. These services are of low intensity and focus mostly on health education rather than skills building. Case managers at study sites help clients connect with primary care physicians for medical issues. Participants from both arms will have access to UC and we will track their use of UC services at each assessment period.

### Fidelity monitoring

We will monitor Peer GLB fidelity throughout the trial. In weekly supervision meetings with peer specialists, the researchers will review attendance logs and fidelity scores, and use group problem-solving strategies to troubleshoot any difficulties. GLB experts will also participate in these supervision meetings on a monthly basis. We will measure three elements of fidelity. Adherence will be captured via fidelity checklists for each group session that specify core session objectives and activities. RAs will complete the checklists for each session based on audio recordings of group sessions. Dose will be calculated as the number of sessions each participant attended during each intervention phase, measured via attendance logs. Participant responsiveness intends to examine clients’ views, experiences and satisfaction with Peer GLB and will be captured using different methods. First, RAs will review trial records to ascertain participants’ completion of intervention activities. Second, clients’ satisfaction with Peer GLB (see assessments below) will be assessed at 6, 12, and 18 months as another indicator of responsiveness. Third, up to 10 focus groups with GLB participants will be conducted once they have completed the 12-month intervention to explore their views and reactions to the intervention. Focus groups will consist of 8–10 participants, last 90 minutes, and will be audio recorded and professionally transcribed. A focus group guide with open-ended questions will be used to elicit participants’ experiences with Peer GLB.

### Procedures to handle treatment contamination

Several measures will be taken to minimize and monitor treatment contamination given that participants’ randomization will occur at the individual level within each study site. Peer GLB sessions will be scheduled at different times from other UC services to minimize interactions between participants. During weekly supervision meetings, peer specialists will be reminded about the experimental nature of the intervention and the importance of not introducing Peer GLB content into other activities at the agencies. To monitor contamination, we will track all participants’ use of UC services in each of our assessment periods. All participants will complete a diffusion questionnaire at each assessment period that contains items asking if they have discussed Peer GLB topics (e.g., self-monitoring) with other participants.

### Measures

Assessments will occur at baseline and at 6, 12, and 18 months post randomization. Bilingual RAs blinded to participants’ treatment condition will administer assessments at the study sites via structured face-to-face interviews. Participants will receive US$25 and a round-trip public transportation reimbursement at each assessment period (see Table 2 for a description of effectiveness study measures).

**Table 2:** Effectiveness trial measures

Type of Variable	Construct	Description	Timeline
Primary Outcome	Weight	Measured in lbs in light indoor clothing without shoes using a digital scale.	B, 6, 12,18
Secondary Outcomes	Anthropometrics	Waist circumference measured to the nearest 0.1 cm with an anthropometric tape, in a horizontal plane 1 cm above the navel in light indoor clothing. Blood pressure assessed on the right arm of participants after they rest quietly in a seated position for at least 5 minutes, using a validated automated sphygmomanometer. Height measured without shoes with an anthropometric tape to the nearest 0.1 cm at entry into the study. Body mass index: will be calculated from measured height and weight (kg/m^2^)	B, 6, 12,18
	Self-efficacy	The Weight Efficacy Lifestyle (WEL) Questionnaire is a 20-item scale that asks people to rate their confidence to resist eating in certain situations on a 10-point Likert scale ranging from 0 (not confident) to 9 (very confident) [90]. The Multidimensional Health Locus of Control (MHLC) is a 24-item scale that measures the extent to which a participant believes s/he has control over various aspects of their physical health [91]. These scales have established psychometrics	B, 6, 12,18
	Physical activity	The International Physical Activity Questionnaire (IPAQ) short form [92] (7 items) asks respondents the number of days per week and the amount of time per day spent in vigorous and moderate activities and walking, during the 7 days prior to the interview. It has established psychometrics, is available in English and Spanish, and is validated for people with SMI [93]. The 6-Minutes Walking Test [94] will be used to measure the distance participants can walk in 6 minutes. This measure has been used in previous health promotion trials with people with SMI [95]. An increase in distance of more than 50 m has been linked to clinically significant reductions in risks for cardiovascular disease [94]	B, 6, 12,18
	Dietary behaviors	The Block Fat Screener and Fruit, Vegetable and Fiber Screener Questionnaires are brief food frequency measures that provide valid assessments of the intake of these foods [96]. Three modules from the 2013 CDC Behavioral Risk Factors Surveillance System Questionnaire: fruit and vegetable, drinks with sugar, and salt/sodium. These modules consist of 11 questions that measure eating behaviors in these areas [97]	B, 6, 12,18
	Recovery	The Recovery Assessment Scale [98] is a 24-item measure that captures different aspects of recovery and produces a total recovery score and scores on 5 subscales	B, 6, 12,18
	Quality of Life	SF-12 [99], a self-report measure available in English and Spanish and validated among adults with SMI that generates two summary scores for physical and mental health-related quality of life [100]	B, 6, 12,18
Moderators	Demographics	Self-reported age, gender, race/ethnicity, education, income, and marital status	B
	Psychiatric medications	Self-reported list of psychiatric medications prescribed. Type and dosage will be recorded	B, 6, 12,18
	Attitudes toward Peer GLB	Modified version of the Client Satisfaction Questionnaire, an 8-item self-report scale available in English and Spanish to assess participants’ attitudes (e.g., satisfaction, acceptability) toward Peer GLB [101]	6, 12, 18
	Mental health	We will use the Revised Behavior and Symptom Identification Scale (BASIS-R) [102], a brief 24-item mental health severity measure designed to assess depression/functioning, difficulty in interpersonal relationships, self-harm, emotional lability, psychotic symptoms, and substance abuse. The BASIS-R is a reliable and valid measure that is sensitive to mental health treatment and has been tested in persons with schizophrenia	B, 6, 12,18
Covariates	Acculturation, barriers to medical care, comorbid medical conditions, service use and alcohol/drug use	Acculturation: nativity, language preference, time in the US, age of migration. The Bidimensional Acculturation Scale [103], a 24-item self-report measure that captures acculturation-related changes in two languages. Barriers to medical care: a list of 11 common factors that may prevent patients from seeking medical care [104, 105]. Comorbid medical conditions: a list of 17 common medical conditions by patient self-report. Service use: we will use established self-reported items from the National Latino and Asian American Study/National Comorbidity Study-Replication to measure participants’ use of mental/physical health services in the previous 6 months. This will help us track use of usual care services in the trial. Substance abuse will be measured using a subset of questions from the Addiction Severity Index (ASI) which assesses frequency of alcohol and drug use [106]	B, 6, 12,18

*B* baseline, *GLB* Group Lifestyle Balance

### Data analysis for effectiveness trial

The distributions of all continuous variables will be checked for normality and transformations will be employed to normalize distributions, if necessary, before applying parametric techniques. All tests will be 2-sided with critical value *α* = 0.05. The distributions of demographic variables in both treatment arms will be examined and, if found to be unbalanced and associated with treatment outcomes, will be included as covariates in all models for those outcomes. A categorical indicator for study sites will be included as a control variable in all models. We will carry out all analyses on an intent-to-treat (ITT) basis using all post-randomization data regardless of whether the participant received or adhered to treatment [74]. Missing data will be imputed, using the MI and MIANALYZE procedures in SAS 9.2 (SAS Institute, Cary, NC, USA). A 2010 national expert panel [75] recommended sensitivity analyses for the impact of missing data via pattern mixture models [76], which we will conduct by investigating robustness of results to perturbations of assumed values for missing data within clinically plausibly ranges.

### Analysis for Aim 1

Hypothesis 1: at 12 and 18 months post randomization, there will be a larger percentage of Peer GLB subjects compared to the UC group who have reached clinically meaningful weight loss (≥5 % weight loss from baseline). This hypothesis will be tested using logistic regression of the dichotomous ≥ 5 % weight loss indicator at 12 and 18 months on treatment and control variables. Additional tests of trend in percent weight loss will be performed across all 3 follow-up times (6, 12, and 18 months) using the generalized linear mixed-effects models described below for secondary outcomes.

### Analysis for Aim 2

Hypothesis 2: at 6, 12, and 18 months post randomization, there will be significant reductions in average weight, waist circumference, and blood pressure and significant improvements in physical activity, dietary behaviors, self-efficacy, recovery, and quality of life in Peer GLB compared to UC. This hypothesis will be tested with a generalized linear mixed-effects model [77]. Separate mixed models, adjusted for control variables, will be fit for different outcomes. Dichotomous outcomes will use a logistic link, while continuous outcomes will use the linear link. The mixed-effects regression model to test this hypothesis will be as follows:

$$ \mathrm{Model}\ (1):{y}_{ijk}={\alpha}_i+{\beta}_{j(i)}+{\tau}_k+{\left(\alpha \tau \right)}_{ik}+\varphi +{\epsilon}_{ijk}, $$

where у_*ijk*_ is the outcome measure for jth subject in ith treatment at time k = baseline, 6, 12, and 18 months; α_*i*_ is the treatment effect (Peer GLB or UC); *β*_*j*(*i*)_ is the random effect of subject j receiving treatment i; *τ*_*k*_ is the effect of time k; (*ατ*)_*ik*_ is the treatment by time interaction; *φ* represents any control variables including an indicator of site; and *є*_*ijk*_ is the experimental error. It is assumed that *β*_*j*(***i***)_ are independent and identically distributed as N(0, σ_b_^2^) for a fixed treatment i, *є*_*ijk*_ are independent and identically distributed as N(0, σ2), and *β*_*j*(*i*)_ and *є*_*ijk*_ are mutually independent. We expect to find a significant treatment-by-time interaction for all outcomes, with Peer GLB participants having better health, recovery, and quality of life than UC participants.

### Analysis for Aim 3

We will explore interactions between treatment and socio-demographics, use of antipsychotic medication, attitudes toward peer specialists, and housing model (housing first versus treatment first) by extending Model (1) above to include interaction terms between subgroup indicators, treatment, and treatment by time. A significant three-way interaction: for example, between gender, treatment, and time would indicate differences in treatment outcome by gender across time. Separate contrasts comparing the treatment effect for men versus women will be formed at each time point to identify and interpret where differences occur. Because of the exploratory nature of these analyses and the low power for identifying interactions coupled with the multiple tests that will be done, caution will be used when reporting all findings [78]. We will use appropriate statistical correction tests (e.g., Bonferroni-Dunn test) to address the possibility of a Type 1 error that can result from multiple testing. We will also include a mediator analysis to explore how the different treatment fidelity elements (e.g., adherence, dose) specified in our conceptual model mediate Peer GLB effects on our primary and secondary outcomes.

### Power analysis

The sample size of 300 was chosen to ensure sufficient power (at least 80 %) of a 2-sided test with level of significance *α* = 0.05 for detecting difference between Peer GLB and UC with respect to the dichotomous primary outcome of clinically meaningful weight loss (≥5 % weight loss) at months 12 and 18. Prior studies have demonstrated a 15 % difference between GLB and UC (i.e., 38 % in GLB versus 23 % in UC) [27]. Assuming 10 % attrition (i.e., *n* = 270) the current study will be able to detect with > 80 % power differences as small as 15 % (e.g., 35 % in Peer GLB versus 20 % in UC) and even be able to detect differences as small as 12.5 % if the clinically meaningful weight loss in the UC group is low (e.g., only 10 % in UC). Attrition of 20 % (*n* = 240) provides power > 80 % to detect differences as small as 16 %. All secondary outcomes are measured on continuous scales. Using the RMASS software (http://www.rmass.org/) to identify detectable treatment effects sizes for repeated measures controlling for site, we find that assuming attrition of 20 % by 18 months, there will be > 80 % power to detect effect sizes as small as 0.25. A meta-analysis [23] found an average difference in percent weight loss between GLB-type interventions and UC of 4.1 % and an approximate standard deviation of weight loss across studies of 8.8 %, indicating an effect size of approximately 0.46. The current study even with a conservative attrition of 30 % (*n* = 210) would be able to detect this effect size with > 95 % power.

### Mixed-methods implementation study (Aim 4)

The aims of this mixed-methods implementation study are to: 1) characterize the outer/inner context of our study sites before, during, and after the introduction of Peer GLB; and 2) identify system-level, organizational-level, and staff-level factors that facilitate or hinder Peer GLB implementation. For this study, we will recruit personnel from the study sites that represent different levels of their organization (e.g., directors, providers). Data for this implementation study will be collected before (Pre-randomized controlled trial (RCT)), during, and after (Post-RCT) we conduct the effectiveness trial (see Table 3).

**Table 3:** Summary of mixed-methods implementation study

Methods	Participants	Projected sample size	Implementation factors examined	Timing		
				Pre-RCT	During the RCT	Post-RCT
Semi-structured qualitative interviews	Agency directors and program managers	6	System-level and organizational-level factors	X		X
Surveys	Direct service providers, including peer specialists	45	Organizational-level and staff-level factors	X		X
Focus groups	Direct service providers and peer specialists	30	Organizational-level and staff-level factors	X		X
Participant observation	Agency directors, program managers, direct service providers, peer specialists, clients	NA	System-level, organizational-level, and staff-level factors	X	X	X

*NA* not applicable, *RCT* randomized controlled trial

### Interviews with directors/program managers

Semi-structured qualitative interviews will be conducted before and after the Peer GLB RCT with directors and program managers to explore system-level and organizational-level factors that could shape the implementation of Peer GLB in supportive housing agencies. Directors will be interviewed as key informants. They will also nominate program managers who oversee health programs at their respective agencies to participate in these interviews. A structured interview guide informed by our conceptual model will be used to elicit information about system-level and organizational-level implementation factors that could influence the implementation of Peer GLB. Interviews will be conducted by a trained RA, last approximately 90 minutes, and will be audio recorded and professionally transcribed.

### Surveys with direct service providers and peer specialists

Self-administered surveys will be conducted before and after the Peer GLB RCT with direct service providers, including peer specialists already employed at these agencies, to examine organizational-level and staff-level implementation factors. The surveys will include measures of staff characteristics (e.g., work tenure) and the following standardized instruments: Organizational Social Context (OSC) to assess organizational culture, climate, and work attitudes [60]; Recovery Self-Assessment (RSA) to capture organizations’ recovery orientation [79]; and the Evidence-Based Practice Attitudes Scale (EBPAS) [69] to examine attitudes towards EBP. Staff, including peer specialists, at each site will complete the survey during scheduled meetings with no upper managers present, after receiving assurances of confidentiality and providing informed consent [60, 65].

### Focus groups with direct service providers and peer specialists

We will conduct focus groups (FGs) with direct service providers, including peer specialists before and after the Peer GLB RCT. Separate FGs for peer specialists and non-peer staff will create a homogenous environment during FG discussions and reduce reporting biases, since some providers may supervise peer specialists. A FG guide informed by our conceptual model will be used to elicit views on the organization’s culture and climate, attitudes toward EBPs and Peer GLB, and experiences implementing Peer GLB. FGs will be conducted after we perform preliminary analyses of the staff surveys to corroborate and/or expand the quantitative findings; a strategy used in small mixed-method studies [80]. Each FG will be composed of 8 participants, last 90 minutes, will be audio recorded, and professionally transcribed.

### Participant observation

Participant observation is a qualitative research method in which researchers observe and/or take part in routine activities and interactions of a group of people in order to better understand their culture [81, 82]. Observations are recorded in a systematic way through written field notes [83]. For this project, participant observation will be conducted to explore UC conditions at the agencies before, during, and after the implementation of the Peer GLB intervention and to examine system-level, organizational-level, staff-level, and client-level factors that could facilitate or hinder the implementation of Peer GLB. Participant observation will also serve as an opportunity to triangulate data collected through the other methods used in this implementation study to better understand the implementation process. Participant observation will focus on agency staff as they carry out their usual activities and, during the implementation of Peer GLB, as they implement this intervention. RAs will seek to observe naturally occurring interactions and conversations among staff and clients that can inform the implementation of Peer GLB and, in later stages that can offer insight into agency context after implementation.

### Quantitative data analysis for implementation study

Given our small sample size of provider surveys (*N* = 45), our quantitative analysis will be descriptive. We will calculate frequencies for categorical variables and measures of central tendencies for continuous variables. Since the agency is the unit of analysis for this implementation study, we will compute agency averages for our quantitative measures noting discrepancies between non-peer staff and peer specialists. We will use bivariate analysis (e.g., *t* tests) to explore differences in agency scores on these measures pre/post the Peer GLB RCT.

### Qualitative data analysis for implementation study

Transcripts will be entered into Atlas.ti (http://atlasti.com/). A grounded theory approach using open, axial, selective and directive coding and the constant-comparative method will inform the analysis [84]. We will use Atlas.ti to mark instances where each code occurs in the data. We will use established procedures to enhance the trustworthiness of our analysis, including using multiple coders, triangulation of data generated from multiple methods and respondents, team de-briefing meetings, prolonged engagement with study sites, development of an audit trail, and member-checking presentations [80].

### Integration of qualitative and quantitative data

We will produce “thick descriptions” [85] of each agency’s outer/inner contexts before and after the Peer GLB RCT by triangulating findings from our different methods. To do this, we will develop a thematic matrix [86, 87] that includes agencies’ characteristics derived from staff/peer specialist surveys (e.g., OSC profiles) and emerging themes from our qualitative data organized by the structural, organizational, and staff levels for each agency pre/post Peer GLB implementation. The matrix will be used to construct detailed organizational case studies from the perspectives of agency leadership and staff noting differences and similarities across contextual levels and time. We will use the thematic matrix to compare side-by-side system-level, organizational-level, and staff-level factors for each agency that were identified as facilitating or hindering Peer GLB implementation. We will note common and unique factors for each agency. This will generate a list of relevant system-level, organizational-level, and staff-level factors and processes organized by level of consensus (i.e., identified by more than one source) and operational salience (i.e., identified as critical for implementation). We will use this list to generate a heuristic model to inform the development of implementation strategies.

## Discussion

Testing and implementing health interventions in community settings is critical for addressing the health disparities faced by people with SMI. This study should make several innovative contributions to the existing literature on the physical health of people with SMI. First, it expands the use of an established intervention to a new service setting not examined in previous health intervention studies in order to reduce access barriers and reach a diverse population with SMI. Second, it contributes new knowledge about the health impacts (e.g., weight, blood pressure, levels of physical activity) of peer-led interventions for people with SMI using a rigorous effectiveness trial design. Third, it addresses the paucity of research examining the health of racially and ethnically diverse people with SMI. Fourth, it provides training and employment for people with SMI enabling them to have a voice in shaping health care. Fifth, it provides an opportunity to explore the effectiveness and implementation of Peer GLB in two commonly used supportive housing models. The hybrid study design will also produce policy-relevant information on the effectiveness and implementation of a novel peer-led healthy lifestyle approach that can inform the allocation of resources in the community to reduce premature mortality among people with SMI. In all, this paper describes the use of a hybrid research design that blends effectiveness trial methodologies and implementation science rarely used when studying the physical health of people with SMI and can serve as a model for integrating implementation science and health disparities research.

## Trial status

Trial recruitment started in June 2015 and is currently open.

## Abbreviations

BMI: body mass index
CVD: cardiovascular disease
EBPAS: Evidence-Based Practice Attitudes Scale
EBP: evidence-based practice
FG: focus group
GLB: Group Lifestyle Balance
ITT: intent-to-treat
OSC: Organizational Social Context
RA: research assistant
RCT: randomized controlled trial
RSA: Recovery Self-Assessment
SMI: serious mental illness
T2D: type-2 diabetes
UC: usual care
US: United States
